# The Psychosocial Burden of HCV Infection and the Impact of Antiviral Therapy on the Quality of Life in Liver and Kidney Transplant Recipients: A Pilot Study

**DOI:** 10.1155/2020/8754247

**Published:** 2020-11-03

**Authors:** Antonella Santonicola, Giancarlo Bilancio, Fabiana Zingone, Laura Donnarumma, Cesare Caputo, Carolina Ciacci

**Affiliations:** ^1^Department of Medicine, Surgery and Dentistry “Scuola Medica Salernitana”, University of Salerno, 84131 Salerno, Italy; ^2^Department of Surgery, Oncology, and Gastroenterology, University of Padua, 35100 Padua, Italy

## Abstract

**Background:**

Therapy with direct-acting antivirals (DAA) for HCV is safe and effective in the liver (LT) and kidney transplant (KT) recipients; however, data on the quality of life (QoL) of patients are scanty. This pilot study is aimed at prospectively evaluating the QoL in LT and KT recipients before and after DAA treatment.

**Methods:**

We prospectively enrolled 17 LT and 11 KT recipients with HCV infection starting a sofosbuvir-based antiviral therapy for 12 weeks. All participants before (T0), 12 (T12), and 24 (T24) weeks after the end of the therapy completed the Short Form Health Survey (SF-36) questionnaire, the Zung Self-rating Depression Scale, and State-Trait Anxiety Inventory (STAI—Y1–Y2).

**Results:**

At T0, LT and KT patients were similar for gender, age, BMI, smoking habits, marital status, mean liver stiffness values at Fibroscan, and HCV genotype distribution (*p* > 0.05). There were no significant differences between the 2 groups in STAI-Y1, STAI-Y2, Zung, and SF-36 scores (*p* > 0.05). At T12, all the participants showed a sustained virological response (SVR). All items of the SF-36 questionnaire improved from the pretreatment to posttreatment period within the LT group, and the 4 domains role-physical, bodily pain, social function, role-emotional, and mental health reached statistical significance (*p* < 0.05 in all cases). On the contrary, in KT patients, there was no significant improvement in SF-36 mean scores compared to at baseline at T12 and T24.

**Conclusions:**

This pilot study suggested that DAA therapy is associated with a significant improvement of the QoL only in LT recipients. Probably, KT recipients did not consider HCV a “central player” in the course of their disease, and HCV eradication did not significantly impact on their QoL.

## 1. Introduction

Chronic hepatitis C virus infection (HCV) globally affects 150–200 million people, ∼3% of the world's population [1]. HCV is one of the leading indications for liver transplant (LT). In the past, in the absence of effective antiviral therapy, HCV reinfection following LT was inescapable and associated with reduced graft and patient survival [2]. About one-third of posttransplant HCV-infected patients developed allograft cirrhosis 5 to 7 years after transplantation [3].

An important interplay also exists between HCV infection and chronic kidney disease (CKD). HCV infection, in fact, associates with cryoglobulinemic glomerulonephritis [4] and membranous types [5]. Furthermore, a high viral load of HCV is associated with an increased risk of developing an end-stage renal disease [6]. In the past, dialysis also represented a significant risk factor for HCV infection due to unsafe injections and the lack of screening of blood transfusions that contributed to the high prevalence of HCV in this population [7]. Dialysis-dependent patients with HCV infection had an increased risk of death and hospitalization and worse quality of life (QoL) scores compared to those without infection [8]. HCV infection influences the outcome of CKD patients also after kidney transplant (KT), increasing the risk of graft loss [9] and *de novo* immune-mediated glomerulonephritis in the graft [10]. In the past decades, the therapy was primarily based on interferon or its long-acting congener (pegylated interferon), with or without ribavirin, with low efficacy rates and frequent adverse events [11]. Treatment of chronic HCV infection has undergone a paradigm change with the introduction of the direct-acting antiviral agents (DAA). Previous studies have demonstrated the high efficacy rates and excellent safety both in LT and KT patients [11–13]. Few studies showed that DAA treatment overall improved the QoL in HCV-infected patients [14, 15].

However, data on quality of life (QoL) after DAA treatment in LT and KT patients are scanty. A recent study demonstrated a significant improvement in QoL 1 year after DAA therapy in a small group of KT recipients [16].

The present study is aimed at evaluating QoL in LT and KT recipients before and after HCV eradication with DAA.

## 2. Materials and Methods

### 2.1. Study Design and Participants

This prospective pilot study included Italian patients (LT and KT recipients) with chronic HCV infection enrolled from Liver and Kidney Transplant Follow-up Centers of the University Hospital of Salerno. The Institutional Review Board of the University of Salerno approved the study protocol. All participants gave their informed consent.

Inclusion criteria were as follows: age from 18 to 65 years, presence of HCV antibodies and HCV-RNA replication, stable renal function in the last six months (defined as the absence of acute kidney injury (AKI) according to Acute Kidney Injury Network criteria [17]), stable levels of immunosuppressive drugs and no graft rejection in the preceding 12 months, and presence of written informed consent.

Exclusion criteria were as follows: contraindications for DAA treatment, renal impairment (estimated glomerular filtration rate, eGFR < 30 mL/min/1.73m^2^), decompensated cirrhosis, chronic hepatitis B virus infection, or human immunodeficiency virus infection. eGFR was calculated by the Chronic Kidney Disease Epidemiology Collaboration Equation (CKD-EPI).

At baseline (T0), we collected demographic information (gender, age, smoking habits, and school degree), anthropometric data (weight, height, and body mass index [BMI]), and prevalence of comorbidities (i.e., hypertension, dyslipidemia, and type II diabetes mellitus). Patients also underwent a physical examination and laboratory tests, including HCV RNA and HCV genotype, alpha-fetoprotein (AFP), immunosuppressive drug levels, and abdominal ultrasound. We also evaluated liver stiffness by FibroScan (Touch 5.02; Echosens; France). They also filled in the Short Form Health Survey (SF-36) questionnaire, the Pittsburgh Sleep Quality Index (PSQI), the Zung Self-rating Depression Scale, and State-Trait Anxiety Inventory (STAI). Patients were enrolled from March 2015 to October 2017. The type of DAA regimen was chosen according to the recommendation of the European Association for the Study of the Liver (EASL) and of the Italian Association for the Study of the Liver (AISF). All the treatments were tailored on patient's characteristics.

All patients started sofosbuvir-based (400 mg/day) antiviral therapy, associated with simeprevir (150 mg/day), daclatasvir (60 mg/day), ledipasvir (90 mg/day), or velpatasvir (100 mg/day). Treatments did not include ribavirin. During the DAA treatment, the levels of immunosuppressive drugs (tacrolimus, cyclosporine, everolimus, and sirolimus) were evaluated once a week; after the end of treatment, evaluation of immunosuppressors was performed every two weeks in the first months and monthly after that.

At 12 (T12) and 24 (T24) weeks after the end of treatment, they performed the same clinical and biochemical evaluation and filled in the questionnaires. We performed FibroScan and abdominal ultrasound again at T24.

Data at T12 and T24 were compared with baseline values to determine the effect of DAA treatment.

### 2.2. Questionnaires

#### 2.2.1. Short Form Health Survey (SF-36) Questionnaire

Health-related QoL was evaluated using the Italian version of SF-36. SF-36 is a generic measure of perceived health status, widely used in medical and health service research, that incorporates behavioural functioning, subjective well-being, and perception of health by assessing some health concepts. The concepts were perception of their physical function, the role-physical (how patients perceive their ability to fulfil their life role physically), bodily pain, general health (overall health and well-being), vitality (how patients perceive their level of “energy”), social function (how patients perceive their ability to participate in social activities), role-emotional (how patients perceive their ability to fulfil their life role emotionally), and mental health (how patients perceive their emotional and psychological well-being). The scores are on scales ranging from 0 to 100, with higher scores reflecting better health [18, 19].

*Zung Self-rating Depression Scale* [20] is a short self-administered survey to quantify the depressed status of a patient. There are 20 items on the scale that rate the four common characteristics of depression: the pervasive effect, the physiological equivalents, other disturbances, and psychomotor activities. There are ten positively worded and ten negatively worded questions. Each question is scored on a scale of 1-4 (a little of the time, some of the time, a good part of the time, and most of the time). The scores range from 25 to 100.

*State and Trait Anxiety Inventory* (*STAI*) [21] has 20 items for assessing trait anxiety and 20 for state anxiety. All items are rated on a 4-point scale (e.g., from “Almost Never” to “Almost Always”). Higher scores indicate the greatest anxiety.

#### 2.2.2. Statistical Analysis

Statistical analyses were performed using SPSS 12.0. Quantitative parametric variables were expressed as mean ± standard deviation, while qualitative parameters were expressed as numbers and percentages. Parametric data were analyzed with repeated measures ANOVA or Student's *t*-test for paired data, as appropriate. Differences were considered statistically significant if *p* < 0.05.

## 3. Results

### 3.1. Baseline Patients' Data

Twenty-eight patients (17 LT and 11 KT recipients) were enrolled.  Table 1 shows their demographic characteristics and selected clinical data at enrollment. There were no significant differences in the two groups for sex, age, BMI, smoking habits, educational and marital status, and HCV genotype distribution (*p* > 0.05). Mean liver stiffness values at Fibroscan were higher in LT than KT patients, although it did not reach the statistical significance (Table 1).

The mean time from transplantation was 12.8 ± 8.2 years in LT and 16.0 ± 8.6 years in KT (*p* = 0.3). Eleven/17 LT patients (64.7%) were on tacrolimus (TAC) monotherapy, 2 (11.8%) on everolimus (EVE) monotherapy, 3 (17.6%) on cyclosporine (CSA) monotherapy, and 1 (5.9%) on mycophenolate-mofetil (MMF) monotherapy. All KT patients were on a combined treatment based on TAC (4 patients, 36.4%), CSA (5 patients, 45.5%), or EVE (patients, 36.4%), in addition to MMF and/or corticosteroids. There were no significant differences between LT and KT patients for alanine aminotransferase (ALT), total bilirubin, gglutamyltranspeptidase (GGT), and aFT at enrollment (*p* > 0.05 in all cases).

#### 3.1.1. QoL and Psychological Assessment

**Figure 1:**
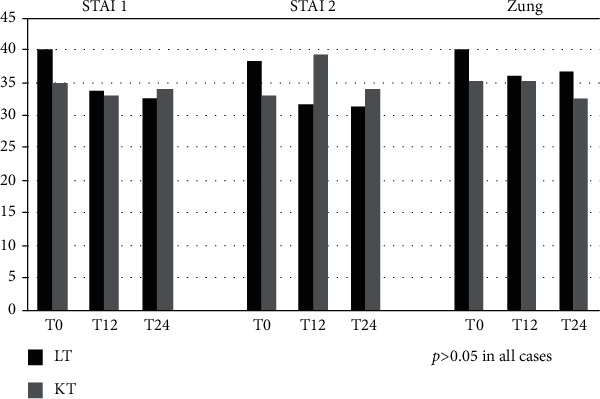
STAI 1, STAI 2, and Zung mean scores at baseline, 12 (T12), and 24 (T24) weeks after the end of DAA therapy in LT and KT patients. Legend: LT: liver transplant; KT: kidney transplant.

All patients (100%) filled in the SF-36, STAI 1 and 2, and Zung questionnaires during their enrollment visit. No differences were found between LT and KT patients in STAI 1, STAI 2, and Zung mean scores (*p* = 0.3 in all cases) (Figure 1).

At SF-36, LT patients reported similar scores compared to KT (*p* > 0.05 in all cases) (Table 2).

LT and KT recipients started sofosbuvir-based antiviral therapy for 12 weeks achieving the complete clearance of HCVRNA at the end of the therapy. No adverse episodes were recorded during the treatment except fatigue (about 20% of the sample).

### 3.2. T12 Evaluation

Twelve weeks after the end of DAA treatment, mean values of ALT significantly decreased both in LT and KT compared to baseline (*p* = 0.01 and *p* = 0.02, respectively). Total bilirubin, GGT, and AFP levels were not significantly different compared to baseline in both LT and KT patients (*p* > 0.05 in all cases). There were no significant modifications of eGFR before and after the treatment, both in LT and KT (*p* > 0.05). All the participants showed a sustained virological response (SVR). Mean liver stiffness values at Fibroscan in LT patients were not significantly different than KT patients (11.4 ± 8.5 vs. 8.5 ± 3.5 kPa). Compared to baseline, mean stiffness values at T24 were significantly lower both in LT and KT patients (paired *t*-test, *p* = 0.007 and 0.02, respectively).

STAI 1, STAI 2, and Zung mean total scores did not significantly differ between LT and KT patients (*p* > 0.05 in all cases) (Figure 1). However, for SF-36, LT reported significantly higher scores than KT patients for physical functioning, role-physical, vitality, and role-emotional (Table 3).

An additional analysis was performed comparing in each group the STAI 1, STAI 2, Zung, and SF-36 mean score at T12 evaluation with those at baseline. There was no significant difference between STAI 1, STAI 2, and Zung mean total scores at T12 and baseline, either in LT or KT patients (*p* > 0.05 in all cases) (Figure 1). In LT recipients, all items of the SF-36 questionnaire improved from the pretreatment to posttreatment period, and the 4 domains role-physical, bodily pain, social function, role-emotional, and mental health reached the statistical significance (paired *t*-test, *p* = 0.04, 0.003, 0.04, and 0.04, respectively).

On the contrary, in KT patients, there was no significant improvement in STAI 1, STAI 2, Zung, and SF-36 mean scores at T12 compared to at baseline.

### 3.3. T24 Evaluation

Twenty-four weeks after the end of DAA treatment, ALT, total bilirubin, GGT, AFP levels, and eGFR were stable and not significantly different compared to those at T12 evaluation in both LT and KT patients (*p* > 0.05 in all cases). STAI 1, STAI 2, and Zung mean total scores did not significantly differ among the two groups (*p* > 0.05 in all cases) (Figure 1). However, in the SF-36 questionnaire, LT patients reported significantly higher scores compared to KT for role-emotional (*p* = 0.001) and general health (*p* = 0.05) (Table 4).

The comparison in each group between the STAI 1, STAI 2, and Zung at T24 evaluation and at baseline did not show any differences both in LT and KT patients (paired *t*-test, *p* > 0.05 in all cases). The SF-36 items physical functioning, general health, vitality, and social function significantly improved in LT patients (paired *t*-test, *p* = 0.04, 0.04, 0.03, and 0.004, respectively) compared to baseline. The SF-36 items did not significantly change from pretreatment to 24 weeks posttreatment in KT patients, with the exception of the item *role*-*emotional* which significantly decreased (paired *t*-test, *p* = 0.04).

## 4. Discussion

The results of this study indicated that after HCV eradication, QoL indices improved in LT, although only for some domains and they did not significantly change in KT patients. Secondarily, we also confirmed the efficacy and safety of sofosbuvir-based regimens in LT and KT recipients. We also reported a significant decrease of mean stiffness values at T24 compared to baseline both in LT and KT patients; however, the sample size is too small to draw absolute conclusions.

Before starting the therapy, LT patients showed similar scores at SF-36 as KT. However, after the HCV eradication, LT patients experienced a “general well-being” which results in improvement both in some physical and mental items of the SF-36. The normalization of liver laboratory tests, the consciousness of HCV eradication, and consequently the impossibility of HCV transmission to their relatives probably played an important role. The psychosocial burden of HCV infection is substantial both in LT and KT recipients; however, KT patients did not experience the same improvement of QoL.

From the early 90s, many studies comparing the QoL in patients with different solid organ transplants have been published [22–27], adding new insights on physical and mental health of this particular group of patients. The novelty of our paper is that we prospectively evaluated QoL before and after the treatment with the new DAA for HCV in a population of LT and KT recipients. We know that the introduction of the DAA had represented paradigm change in the therapy of chronic HCV infection. Some authors focused on the QoL after this therapy in other class of patients [14, 28]; however, the topic of QoL and HCV therapy in solid organ transplants was poorly assessed. We evaluated QoL of LT recipients because HCV infection was the main cause of liver transplantation in our country. Secondly, we chose to compare them to a group of KT recipients considering the high number of patients who were infected by HCV due to dialysis in the past.

A recent study evaluated QoL in 16 KT recipients [16] before, at the end of DAA treatment, and 1 year after. The authors revealed a consistent improvement in the emotional domains, while there were no significant changes in physical domains. Our results did not confirm these data, showing a decrease of the *role-emotional* at T24 evaluation. This could be explained by the different follow-up periods and the small population enrolled in both the two studies.

Several hypotheses could explain the lack of improvement of QoL in KT recipients. They may consider HCV a “marginal” rather than a “central figure” in the natural course of their disease. Moreover, they often expressed concerns about the safety of DAA therapy on renal function that could contribute to a less impressive improvement of their QoL.

The main limitation of this study is the small sample size. Firstly, our work was a pilot study; secondly, it considered a population of LT and KT recipients who were homogeneous for geographic origin (Campania, South of Italy), education levels, and marital status [29, 30] that could affect QoL. The small number of patients may be also the consequence of the accurate selection of patients. In fact, also the few studies that previously evaluated this topic enrolled a small number of patients [16, 31]. Furthermore, the enrolled patients were also homogenous for the quality of follow-up as they were regularly followed by Liver and Kidney Transplant Follow-up Centers of the University Hospital and had scheduled clinical evaluations, laboratory tests, and instrumental examinations according to a rigorous protocol. We cannot exclude that the strict follow-up regimen may have contributed to excellent adherence rates to HCV therapy.

Data support the need for long-term studies to evaluate whether the early HCV eradication both in liver and kidney transplants will change the course of the disease, reducing the incidence of complications.

## 5. Conclusion

Life expectancy after the transplant is increasing [32], likely due to advances in many surgical and medical aspects. The improved survival after liver or kidney transplant stresses the importance of focusing also on psychosocial aspects that, as well as physical conditions, profoundly impact on the QoL of these patients. The psychosocial burden of HCV infection probably will be eliminated by DAA therapy, but other social aspects could play a role in the patient's QoL and perception of the disease.

## Figures and Tables

**Table 1:** Demographic characteristics of LT and KT patients. Data were expressed as percentage (%) or mean ± standard deviation (SD).

	LT patients *N* = 17	KT patients *N* = 11	*p*
Males (%)	10 (58.8)	6 (54.5)	0.8
Age	67.3 ± 11.4	60.7 ± 11.6	0.1
Smoke (%)	4 (23.5)	3 (27.3)	0.8
Alcohol (%)	3 (17.6)	4 (36.4)	0.2
Married (%)	15 (88.2)	10 (90.9)	0.8
Low school degree (%)	10 (58.8)	7 (63.6)	0.8
Weight (kg)	72.1 ± 7.6	67.4 ± 12.2	0.2
BMI (kg/m^2^)	26.4 ± 3.1	26.2 ± 3.5	0.8
eGFR (mL/min/1.73m^2^)	69.1 ± 20.8	60.2 ± 15.7	0.2
Liver stiffness (kPa)^∗^	12.2 ± 9.5	8.9 ± 3.9	0.3
Genotype			0.4
1a/1b	1/11 (5.9/64.7%)	2/8 (18.2/72.7%)	
2	4 (23.5%)	1 (9.1%)	
3	1 (5.9%)		

LT: liver transplant; KT: kidney transplant; eGFR: estimated glomerular filtration rate.

**Table 2:** The eight-item SF-36 scores in LT and KT patients at enrollment. Data are expressed as mean ± standard deviation (SD).

SF-36 components	LT patients *N* = 17	KT patients *N* = 11	*p*
Physical function	53.6 ± 30.8	64.3 ± 21.5	0.4
Role-physical	39.3 ± 47.7	50 ± 41.8	0.6
Bodily pain	58.8 ± 30.7	63.4 ± 32.2	0.7
General health	40.6 ± 30.6	50.3 ± 28.2	0.5
Vitality	51.1 ± 21.8	64.2 ± 28	0.3
Social function	59 ± 23.5	62.4 ± 38.3	0.8
Role-emotional	45.1 ± 44.6	49.8 ± 40.9	0.8
Mental health	54.9 ± 19.9	65.3 ± 29.7	0.4

LT: liver transplant; KT: kidney transplant.

**Table 3:** The eight-item SF-36 scores in LT and KT patients 12 weeks (T12) after the end of DAA therapy. Data are expressed as mean ± standard deviation (SD).

SF-36 components	LT patients *N* = 17	KT patients *N* = 11	*p*
Physical function	81.0 ± 24.5	46.4 ± 29.0	0.02
Role-physical	82.5 ± 37.4	28.6 ± 48.8	0.02
Bodily pain	81 ± 28.5	67.1 ± 69.4	0.4
General health	60.5 ± 25.8	40.4 ± 30.8	0.2
Vitality	72.5 ± 18	47.1 ± 26.9	0.03
Social function	88.6 ± 20.1	71.4 ± 32.9	0.2
Role-emotional	93.3 ± 21.2	14.4 ± 17.6	<0.001
Mental health	81.6 ± 16.02	64 ± 26.8	0.1

LT: liver transplant; KT: kidney transplant.

**Table 4:** The eight-item SF-36 scores in LT and KT patients 24 weeks (T24) after the end of DAA therapy. Data are expressed as mean ± standard deviation (SD).

SF-36 components	LT patients *N* = 17	KT patients *N* = 11	*p*
Physical function	79.5 ± 18.6	66.2 ± 32.0	0.2
Role-physical	68.2 ± 41.9	50 ± 48.2	0.4
Bodily pain	74.3 ± 28.3	64.5 ± 26.8	0.4
General health	67.2 ± 24.9	35.7 ± 38.5	0.05
Vitality	68.5 ± 20	56.4 ± 27.3	0.3
Social function	86.1 ± 19.9	78.1 ± 24.7	0.4
Role-emotional	81.7 ± 34.6	32.6 ± 24.9	0.001
Mental health	74 ± 21.4	56.5 ± 28.7	0.1

LT: liver transplant; KT: kidney transplant.

## Data Availability

Data available on request, contacting the corresponding author Carolina Ciacci.
